# Longitudinal, label-free, quantitative tracking of cell death and viability in a 3D tumor model with OCT

**DOI:** 10.1038/srep27017

**Published:** 2016-06-01

**Authors:** Yookyung Jung, Oliver J. Klein, Hequn Wang, Conor L. Evans

**Affiliations:** 1 Wellman Center for Photomedicine, Harvard Medical School, Massachusetts General Hospital, CNY149-3, 13th St, Charlestown, MA USA

## Abstract

Three-dimensional *in vitro* tumor models are highly useful tools for studying tumor growth and treatment response of malignancies such as ovarian cancer. Existing viability and treatment assessment assays, however, face shortcomings when applied to these large, complex, and heterogeneous culture systems. Optical coherence tomography (OCT) is a noninvasive, label-free, optical imaging technique that can visualize live cells and tissues over time with subcellular resolution and millimeters of optical penetration depth. Here, we show that OCT is capable of carrying out high-content, longitudinal assays of 3D culture treatment response. We demonstrate the usage and capability of OCT for the dynamic monitoring of individual and combination therapeutic regimens *in vitro*, including both chemotherapy drugs and photodynamic therapy (PDT) for ovarian cancer. OCT was validated against the standard LIVE/DEAD Viability/Cytotoxicity Assay in small tumor spheroid cultures, showing excellent correlation with existing standards. Importantly, OCT was shown to be capable of evaluating 3D spheroid treatment response even when traditional viability assays failed. OCT 3D viability imaging revealed synergy between PDT and the standard-of-care chemotherapeutic carboplatin that evolved over time. We believe the efficacy and accuracy of OCT *in vitro* drug screening will greatly contribute to the field of cancer treatment and therapy evaluation.

Tumor heterogeneity is thought to be a major factor in poor cancer treatment response and the development of treatment-resistant disease^1^. In ovarian cancer, for example, although 85% of patients will initially experience a complete therapeutic response, a significant majority of patients eventually succumb to recurrent, treatment-resistant metastatic ovarian cancer^2^,^3^. This high rate of recurrence leads to poor quality of life and an overall low 5-year survival rate of 30%. However, ovarian cancer is one of the most treatable malignancies when detected early, with Stage I patients having greater than a 95% survival rate^4^. The difficulty in finding lasting treatments for late-stage patients is thought to stem from the highly heterogeneous nature of metastatic ovarian cancer, which exhibits not only widespread intra-and intertumoral genetic diversity, but also phenotypic and microenvironmental diversity^4^. In particular, a subpopulation of ovarian cancer cells are thought to have tumor-initiating or stem-like properties that allow even a small set of surviving cells to repopulate a patient with tumors^5^,^6^.

Much of this cellular heterogeneity is unfortunately lost when tumor cells are plated on standard plastic culture dishes, which have stiff surfaces and lack biologically-relevant cell-cell and cell-matrix interactions. Three-dimensional *in vitro* tumor cultures restore many of these important variables, and have been shown to replicate many features of ovarian tumors found *in vivo*^7^. 3D *in vitro* culture models are of particular significance in studies of therapeutic response in ovarian cancer as their size and complexity are similar to that of ovarian metastatic lesions. Metastatic ovarian cancer studs the surfaces within the peritoneal cavity and is composed of small tumor nodules that range in size from small avascular lesions a few hundred microns in diameter to larger occult lesions several centimeters wide. The standard-of-care surgical resection received by nearly all ovarian cancer patients is considered successful if the remaining metastatic lesions are less than 1 cm in diameter. 3D *in vitro* ovarian cancer cultures that mimic these small residual and often avascular lesions are considered highly important as they model the target tumor dimensions of interest for the majority of therapeutics currently under development.

Despite their strengths, 3D cultures can prove difficult to interrogate accurately; disaggregating 3D multicellular spheroid cultures into individual cells can enable high-throughput analysis, but eliminates important spatial information. Traditional techniques that have been used to monitor treatment response include fluorescence imaging. The most commonly used method is the LIVE/DEAD Viability/Cytotoxicity Assay, which labels the live and dead cell populations with different fluorophores in order to differentiate and quantify these two cellular states^7^,^8^,^9^. High-content imaging of 3D cultures with fluorescent markers can successfully map viability and treatment response in small (<200 μm diameter) spheroids^7^, but the majority of these methods are limited to a single timepoint. When investigating large spheroid cultures, which contain hypoxic and acidic compartments known to influence treatment response, fluorogenic methods can fall short. Fluorescence imaging, even when using multiphoton microscopy, suffers from relatively low penetration depth, limiting the ability to assess treatment response in spheroids’ hypoxic microenvironments^9^. More problematic is the limited uptake and penetration of fluorescent cell viability reporters themselves into multicellular spheroid cultures; many reporters penetrate only a few hundred micrometers, and their distribution throughout the spheroid can be non-uniform, making accurate treatment response assessment difficult. The accuracy of viability markers can also be perturbed by cellular factors: for example, the cleavage rate of the non-fluorescent calcein AM ester into the fluorescent live-cell marker calcein can be modulated by the concentration of intracellular esterases. Overall, a lack of accurate cellular viability assays can limit the utility of 3D cultures in investigating and optimizing cancer treatments, especially for complex and heterogeneous *in vitro* systems that model challenging cancer microenvironments.

In order to better quantify therapeutic response in 3D culture systems, advanced optical imaging methods have been developed with the goal of overcoming these limitations. The use of multiphoton microscopy methods, for example, can improve the depth of imaging within 3D cultures by a factor of two or three, but is still limited by the need for fluorescent labels. Optical coherence tomography (OCT), an interferometry-based optical ranging method, advantageously is capable of label-free imaging at depths exceeding several millimeters, enabling large-scale (millimeters) cross-sectional morphological views of tissue structures with submicron-level imaging resolution^10^,^11^. Analogous to ultrasound, OCT detects photons scattered off surfaces in tissue, with the image contrast originating from variances in the sample’s refractive index^12^,^13^. Because of its high penetration depth (usually a few millimeters), relatively high resolution (~microns), and fast scanning speed, OCT can be used to perform long-term, high-throughput structural imaging of 3D culture systems^9^,^14^,^15^.

OCT has been proposed as a quantitative method for monitoring and evaluating treatment response *in vitro*^16^,but there have been few studies that have attempted to quantify and correlate OCT image-based metrics with standard assays. There have been OCT-based metrics for characterizing tissue properties, such as OCT signal slope and spatial standard deviation^17^, fractal dimension analysis^18^, as well as automatic microstructural quantification^19^. However, the properties of images acquired from tissue tend to be different enough from those acquired from 3D model nodules to make these methods inaccurate when applied *in vitro*. This arises from the nature of the model systems: *in vitro* model tumor nodules are small clusters of cells largely without organized structure, considerable stroma, or surrounding tissue environments. In this study, we carried out OCT treatment response analysis specific to 3D *in vitro* ovarian cancer spheroids and benchmarked these results against the accepted LIVE/DEAD Viability/Cytotoxicity Assay. We found that OCT was able to surpass many of the limitations of this fluorogenic assay, providing metrics on treatment outcome while Live/Dead response remained flat and unchanged. To further demonstrate the advantage of OCT, a series of experiments were carried out to track treatment response over time, using OCT for the continuous monitoring of *in vitro* cultures. Unlike fluorogenic assays that measure a single timepoint, OCT instead revealed the evolution of synergy in a photodynamic therapy/chemotherapy combination regimen. Based on these results, OCT methods have the ability to play an important role in treatment evaluation in the field of cancer research.

## Results

### OCT metrics match Live/Dead Assay response in small tumor spheroids

3D *in vitro* ovarian cancer cultures were developed using OVCAR5 cells, which can generate large, complex three-dimensional nodules ranging from 400–700 μm in diameter^9^. This model mimics the phenotypic presentation of the myriad of small avascular metastatic ovarian cancer lesions that remain following “debulking” surgical intervention. To first validate OCT measurement outcomes, OCT imaging was compared against the Live/Dead assay in small OVCAR5 spheroids where the uptake of the Live/Dead fluorescent agents is not a limiting factor^7^. Cytotoxic agents typically have limited uptake and penetration, and are usually not uniformly distributed within model tumor nodules. To avoid these potential limitations in this validation step, we employed a photodynamic therapy regimen using 5-aminoleuvaneutic acid (5-ALA), which has been shown in past studies to be uniformly distributed throughout the cells within 3D cancer model^16^. 5-ALA is a naturally occurring small molecule used in the heme biosynthesis pathway. When delivered exogenously at micromolar concentrations, the heme biosynthesis pathway can be overwhelmed, leading to the generation of excess protoporphyin IX (PpIX), a naturally occurring photosensitizer, within mitochondria^20^. 5-ALA was particularly useful for this study as its uptake and conversion to PpIX occurs throughout spheroids at micromolar and higher incubation concentrations^21^. Moreover, as PpIX photodynamic therapy triggers cellular death via mitochondrial damage, there is no change in cellular esterase activity upon treatment.

**Figure 1:**
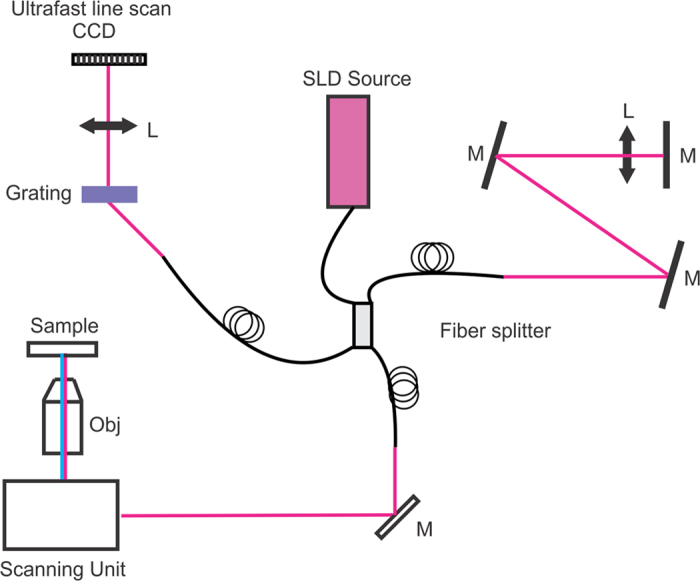
Diagram of the developed OCT system. A broadband superluminescence light source was coupled into a Michelson interferometer. The sample arm provides light to the microscope imaging system, while the reference arm is designed to have the same optical path length as the sample arm. Interference between the sample and reference arms is collected through a home-made spectrometer. Obj: objective lens, L: lens; SLD: superluminescence diode; M: mirror.

**Figure 2:**
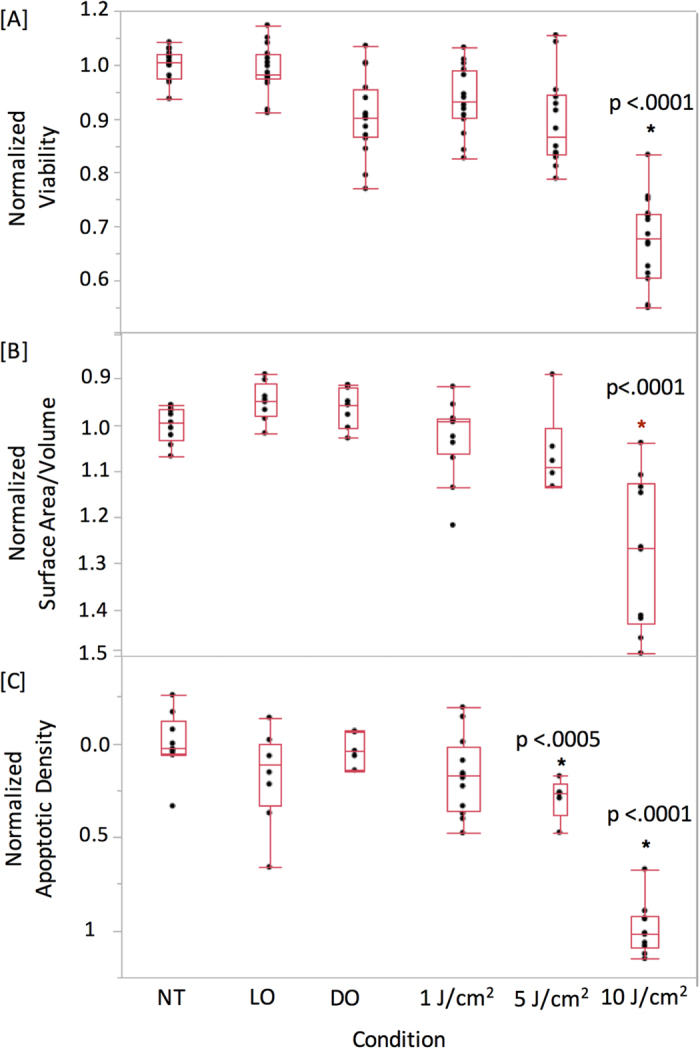
Validation of OCT metrics with the fluorogenic Live/Dead viability assay. [**A**] OCT imaging data from the same dishes was processed to calculate the normalized surface-area-to-volume [**B**], or the normalized apoptotic density [**C**]. Normalized viability was calculated by computing the live/live + dead value and normalizing to the no-treatment control. Three samples were used for each measurement (N = 3). Normalized surface-area-to-volume was calculated using segmentation-based analysis, with each value normalized to the no treatment control. No-treatment control was normalized to a value of unity, corresponding to intact spheroids. Normalized apoptotic density was calculated via segmentation-based analysis and normalized to the no-treatment control. For comparison with the Live/Dead assay and for clarity of presentation, the OCT-derived surface-area-to-volume and apoptosis plots are displayed with an inverted y axis. Both apoptosis and nodular disruption indices closely match the treatment response measured via the Live/Dead viability assay. The 5 J/cm^2^ response in Fig. 2C is statistically significant when compared pairwise with the NT group (p = 0.0003), the LO group (p = 0.032), and the DO group (p = 0.0013). NT: no treatment control; LO: light only control; DO: drug only control.

Spheroids treated with 1 μM of 5-ALA were exposed to a range of 635 nm light doses to trigger cellular death. Each well was then imaged 24 hours later with a custom-build OCT system (Fig. 1) to collect image volumes for quantitative analysis. Following OCT, wells were then treated with the Live/Dead reagents and imaged out using an automated confocal microscope (Olympus FV1000 configured on an inverted IX81 base). In this way, both data sets were generated from the same spheroid cultures. The viability of each well was determined using the Live/Dead assay coupled with image analysis (Fig. 2A).

As cell death can occur via both apoptosis and necrosis following ALA/PpIX photodynamic therapy, OCT images were analyzed to generate metrics derived from cell death processes. Both apoptosis and necrosis lead to the loss of cell-cell adhesion, which in the case of 3D nodules and tissue, causes structural disruption and fragmentation^7^,^15^,^20^,^22^. To quantify this change, a metric was calculated based on surface-area-to-volume ratio. OCT data were first processed by measuring the surface area and volume of each computationally identified object within a nodule^15^. The ratio of these individual objects was averaged for each data set and then normalized to the baseline no-treatment control. The surface-area-to-volume disruption index closely matches the results of the Live/Dead viability assay, with statistically significant results observed for only the 10 J/cm^2^ light dose (p < 0.0001) (Fig. 2B).

While spheroid disruption is an indirect measure of cell death, OCT can also be used to quantify apoptosis directly. During apoptosis, cells undergo a series of processes that package cellular contents in small dense vesicles creating a local index of refraction mismatch; these vesicles thus scatter light strongly and provide unique contrast *in vitro.* As has been shown in numerous reflection and OCT studies^14^, the apoptotic process therefore leads to the appearance of strongly scattering bodies within 3D tumor nodules that can be detected and enumerated. The identity of these strong scattering bodies has been confirmed in past studies by us to only arise from apoptotic cells *in vitro*^14^.

The same image data collected above was re-processed using an image analysis routine that enumerated the volume of individual, highly scattering clusters of apoptotic bodies to calculate a normalized apoptotic density metric (Fig. 2C). Control experiments using the Live/Dead kit were used to independently verify the accuracy of the image analysis routine. Interestingly, processing the data for apoptosis indicated that the 5 J/cm^2^ treatment dose gave a statistically significant change in the number of apoptotic bodies per nodule (p = 0.0003), an observation not captured by the Live/Dead assay. The degree of apoptosis triggered by PDT was measured to be highly consistent with low variability from nodule-to-nodule and well-to-well, indicating that the treatment led to well-defined levels of programmed cell death. When measuring surface-area-to-volume, on the other hand, there was greater spread in the data indicating variability in the measured cellular viability. This higher degree of variability is confirmed by examining a similar variance in the LIVE/DEAD viability data (Fig. 2A). Taken along with the apoptosis results, this data set indicates that the photodynamic treatment response is heterogeneous from nodule-to-nodule, matching known PDT outcomes in this 3D model system^8^,^9^. We hypothesize that the less heterogeneous apoptosis outcomes arise due to the nonlinear nature of the PDT response: there is always a distribution in the degree of PDT-induced radical damage across cells, but only cells within a narrow portion of that distribution will accumulate enough damage to reach the threshold necessary to trigger apoptosis. This nonlinear narrowing may lead to the observed low heterogeneity in the apoptotic response.

It is worth noting that Fig. 2 indicates a difference in the threshold PDT dose that lead to statistically significant changes in apoptosis (5 J/cm^2^) and surface-volume-to-area ratio (10 J/cm^2^). This result suggests that, at a dose of 5 J/cm^2^, numerous cells are undergoing the apoptotic process, but the treatment dose is likely not high enough to cause levels of cell death where cell-cell adhesions are lost in high numbers. The higher treatment dose of 10 J/cm^2^, on the other hand, causes significantly greater cell death, leading to a loss of adhesions that can be quantified via surface-area-to-volume changes. These differences likely follow from the reactive oxygen species-driven nature of PDT, where therapeutic outcomes are well known to occur at threshold levels of treatment.

### Evaluating Treatment Response with OCT metrics overcomes the limitations of fluorogenic assays

**Figure 3:**
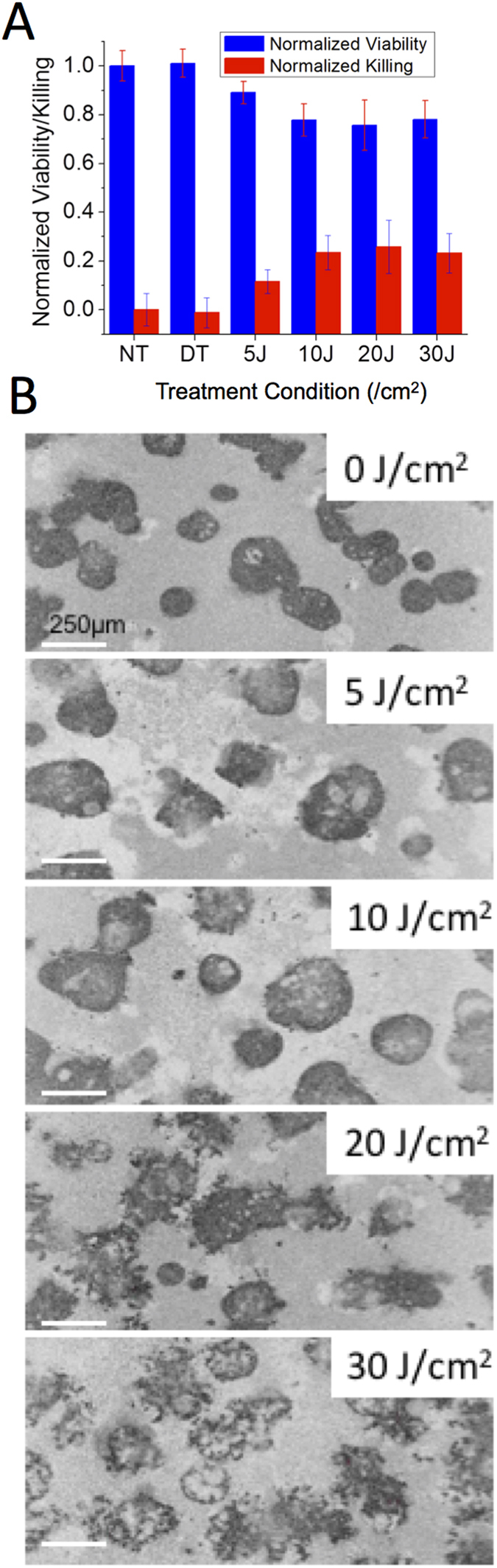
The Live/Dead assay underreports cell death and viability when used in conjunction with the lysosomal photosensitizer EtNBS. Both viability and cell killing measurements plateau at 10 J/cm^2^ and beyond (**A**), even though spheroids clearly undergo greater disruption and apoptosis (**B**). Whiskers represent the standard error.

While fluorogenic assays such as the Live/Dead method were found compatible with mitochondrial ALA therapy in small spheroids, other mechanisms of cell death can significantly alter assay results. Cellular death via lysosomal disruption is one such condition, where esterases initially confined to lysosomes enter the cytosol and can perturb measurements that rely on the cleavage of ester bonds. This limitation can be observed in Fig. 3. OVCAR5 spheroids were grown for 10 days, after which they were treated with EtNBS photodynamic therapy across a range of light doses. EtNBS, which localizes primarily to lysosomes, causes lysosomal rupture and the initiation of apoptotic cell death upon photo-illumination with 660 nm light. When using the Live/Dead assay (Fig. 3A) the reported cellular viability plateaued at 10 J/cm^2^ of treatment light, even though spheroids displayed increased disruption and cellular death via apoptosis at higher treatment doses (Fig. 3B). Investigation of the calcein green and ethidium homodimer imaging data revealed that while the “dead” ethidium signal increased with treatment dose as expected, the “live” calcein green signal remained uniformly high at all light doses, leading to a significant underreporting of treatment efficacy. The high calcein signal observed is clearly in error, as the ALA/PpIX experiments above, as well as prior PDT studies^7^,^23^, consistently showed this live signal to decrease at greater levels of cellular death.

**Figure 4:**
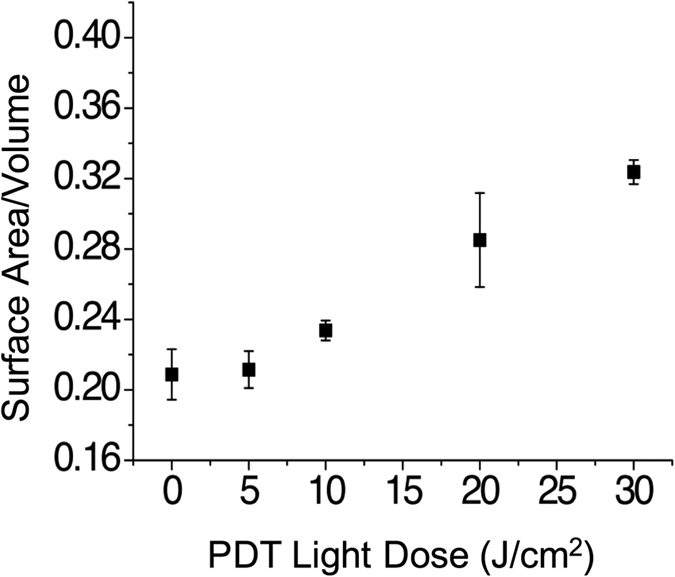
OCT image metrics report treatment response. OCT data acquired from the same samples used to generate Fig. 3 demonstrate that OCT reliably reports changes in treatment response at increased therapeutic light doses.

The same spheroids treated with EtNBS-PDT and quantified with the LIVE/DEAD assay were imaged using OCT, with OCT data processed to generate the surface-area-to-volume index described above. As shown in Fig. 4, the therapeutic response of the *in vitro* 3D tumor spheroids can be successfully tracked at all light doses, with an increase in the surface-area-to-volume ratio observed as cells undergo apoptosis in response to EtNBS-PDT. These results indicate that quantitative morphology-based OCT analysis can readily reveal therapeutic efficacy, even when molecular assays such LIVE/DEAD.

### OCT can reveal time-dependent changes in treatment response and synergy

One key advantage of OCT-based therapeutic response monitoring is that imaging data can be acquired longitudinally, without any need to perturb or alter the sample. This differs from the majority of molecular and fluorescent assays, which are typically terminal and single-timepoint and terminal. Once applied, these assays either alter samples or cannot be used at later timepoints. For example, cell death markers, such as ethidium homodimer and propidium iodide, bind to DNA and permanently alter cells and their growth *in vitro* (data not shown). In contrast, OCT-based viability assays can follow the real-time dynamics of treatment response in the same *in vitro* samples over hours, days, and even weeks if using automated imaging systems. The ability to track the fate of individual cells or spheroids opens up the possibility of capturing rare events or opportunistic therapeutic windows following initial treatment.

To demonstrate this ability, OCT was used to monitor the response of *in vitro* spheroids following combination therapy. Prior studies using a hydroxyl chain derivative of EtNBS, EtNBS-OH, showed that this molecule was capable of triggering the unpacking of tumor spheroids in culture, leading to structural alterations that may reoxygenate the spheroid core and allow for the influx of otherwise penetration-limited drugs^7^,^24^. As the change in spheroid structure is transient, it was proposed that there may be a window during which combination therapy can be optimized.

**Figure 5:**
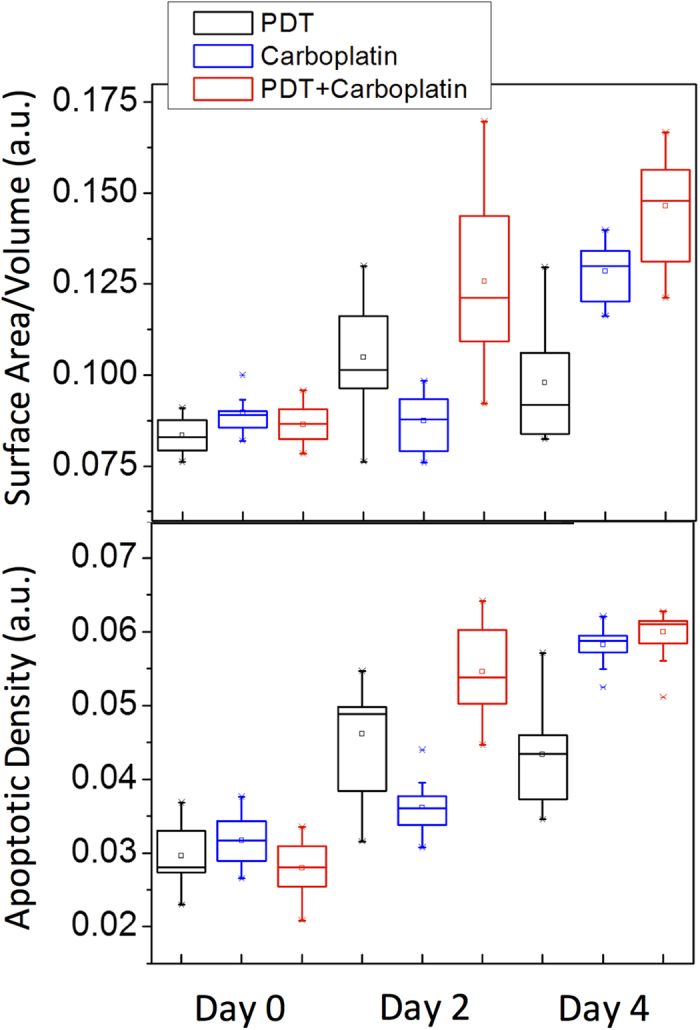
OCT reveals treatment synergy in the days following combination EtNBS-OH PDT and carboplatin therapy. Treatments were initiated after ten days of growth (Day 0). Three samples were used for each measurement (N = 3). The surface-area-to-volume ratio shows synergy between PDT and carboplatin at both two and four days post-treatment. Interestingly, synergy is observed at only two days post-treatment when the OCT data is analyzed for apoptotic density.

Spheroid cultures were plated and allowed to grow for a total of ten days prior to the beginning of treatment. PDT was performed using 500 nM of EtNBS-OH with a 1.5-hour incubation time and 10 J/cm^2^ of light exposure. For chemotherapy, carboplatin was administered at a concentration of 200 μM and a 72-hour incubation time. As shown in Fig. 5, for PDT treatment alone, the maximum killing efficacy was found two days post-treatment. In contrast, with carboplatin chemotherapy, there is an increasing trend of nodular disruption starting from the day of treatment (surface-area-to-volume ratio of ~0.085) to four days post-treatment (surface-area-to-volume ratio of ~0.13)^23^. Interestingly, when combining EtNBS-OH PDT with chemotherapy, synergy was initially observed based on the OCT data, which is consistent with previous study results showing a synergistic enhancement of the carboplatin efficacy in combination with spheroid-disrupting PDT^7^. Moreover, this synergistic window evolved over time, reaching a maximum at two days post-therapy and decreasing thereafter.

**Figure 6:**
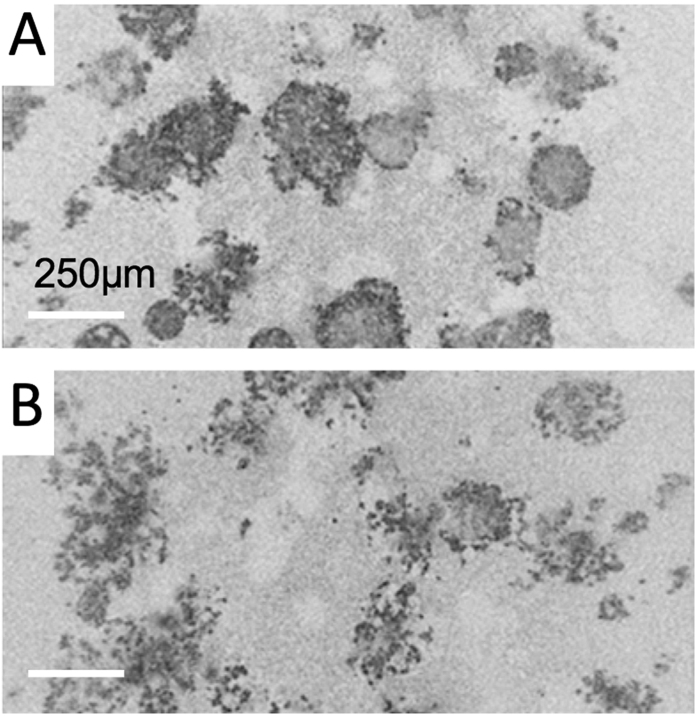
*En face* OCT images of *in vitro* spheroids treated with carboplatin (**A**) or PDT-carboplatin combination therapy (**B**) on day 4. Spheroids treated with carboplatin only show largely structurally intact nodules that feature apoptotic cells on the spheroid periphery. Cultures treated with the combination therapy, on the other hand, show structurally degraded nodules with widespread apoptosis.

Measurements of apoptosis following therapy not only correlated well with the nodular disruption analysis, but also provided complementary insight into the dynamic evolution of treatment response. As shown in Fig. 5, comparing with the surface-area-to-volume ratio results, the overall trend is the same for each separate treatment. However, when the combination treatment results are analyzed, the apoptotic density metric only indicated synergy at two days post-treatment, but not at the four day post-therapy timepoint. The reason for this difference can be gleaned from the comparison of the *en face* OCT images in Fig. 6. While carboplatin treatment causes widespread apoptosis 72 hours following therapy, cells within each spheroid largely underwent apoptosis in place. Individual spheroids can be observed with apoptotic cells lining the periphery of the nodule. (Figure 6A) Many of these same spheroids, however, do not contain apoptotic cells within their core, indicating that cells within the spheroid were shielded from therapy as previously reported^9^. In contrast, combination therapy with EtNBS-OH-PDT leads to structural breakdown of the spheroid, such that apoptotic bodies are found throughout entire disrupted nodules (Fig. 6B). The difference in total number of apoptotic bodies between carboplatin treatment alone and EtNBS-OH-PDT/carboplatin therapy is small, however, explaining the lack of observed synergy via apoptotic density observed four days post-treatment. As the level of nodular disruption is greater in the combination therapy arm, synergy in this metric is observed at all timepoints. The differences between these metrics is important, as they inform on the mechanisms of treatment response and the pattern of cellular death following therapy. These different quantitative metrics derived from the OCT images provide complementary information for evaluating treatment efficacy.

## Discussion

Assessment of treatment response in 3D tumor models is routinely carried out using fluorescence-based assays. These approaches, however, can be significantly limited by both optical and probe penetration depth, largely limiting quantitative measurements of tumor spheroids. These assays are typically terminal in nature and often are limited to single timepoint measurements. Moreover, fluorescence-based viability assays can easily misreport treatment response, especially when used to assess high-dose therapeutics. To overcome these limitations, we developed and validated an OCT imaging approach that can noninvasively and continuously visualize 3D cultured tumor nodules with penetration depth of several millimeters and report treatment response with high sensitivity. Through quantitative analysis of OCT images, we have demonstrated the consistency between the results derived from OCT images with the Live/Dead fluorescence imaging results. We also found synergy between PDT and chemotherapy regimens, pointing to the ability to identify critical temporal windows where combination regimens might be optimized.

An interesting outcome of this study was the observed difference between the surface-area-to-volume ratio and the measurement of apoptotic density within spheroids. Though the metrics showed similar patterns of treatment efficacy when evaluating the combination treatment regimen, the difference between the metrics indicated a discrepancy that required further evaluation. Unlike traditional plate reader fluorogenic assays, the time-lapse OCT data could be re-mined and interpreted long after the experiment. Like their counterparts within tumors, cells within the core of *in vitro* spheroids have been observed to escape therapy, either via the poor penetration of chemotherapy drugs^25^ or through the presence of certain microenvironmental factors including hypoxia^26^. This is particularly problematic in ovarian cancer, where the high degree of intratumoral heterogeneity has been linked to recurrence following initially successful therapy. The differences between the disruption and apoptosis metrics could be interpreted via the 3D OCT tomograms, pointing out the importance of the combination regimen and its ability to disrupt spheroid structure: by unpacking the spheroids first, cells within the spheroid cores could be more effectively killed.

The ability to analyze OCT treatment response data sets can be carried further beyond the two methods discussed in this study. OCT volumes can be mined for specific treatment response patterns, both spatially and temporally, to glean additional treatment response information. Segmentation algorithms, for example, could be applied to separate out apoptotic response in the periphery versus the core of spheroids. Similarly, individual spheroids can be tracked with their specific therapeutic response quantified over time to capture the heterogeneous treatment response between tumor nodules. The ability to mine OCT data sets makes this method highly flexible, as numerous specific questions can be probed from a single data set once preliminary analyses have been carried out.

As 3D nodules are recognized to be highly important in the development of new therapeutics, and have been highlighted by the National Institutes of Health as critical model systems for therapeutic studies, we believe this OCT method will be of use to many researchers. This is especially true for studies of large and complex model systems, as well as for high-throughput 3D model therapeutic screening studies. The OCT imaging method is compatible with multiwell plates and motorized stages, enabling time-lapse acquisition of OCT treatment response data from thousands of model tumor nodules in a single experiment. The data analysis routines, coded here in Matlab, can also be reprogrammed to take advantage of modern GPU’s, significantly accelerating image analysis speed. While this method is not intended for tissue or animal models, it is also likely applicable to cultured tissue and slice tissue culture systems.

Viability analysis with OCT has the capacity to become a powerful utility for high-throughput, high-content drug screening in 3D *in vitro* cultures, and could greatly enhance the development of therapies for ovarian cancer and other malignancies. Future steps require not only the creation of automated OCT time-lapse imaging systems, but also step-by-step validation of new metrics with traditional, gold-standard methodologies. As this toolkit continues to develop, it is expected to aid in the identification and optimization of improved therapeutic strategies. Especially exciting is the future application of OCT methods to novel small molecule and biologic therapies for both drug development as well as the construction of personalized therapy regimens.

## Methods

### Cell culture and 3D culture

OVCAR5 human epithelial cells were obtained from Fox Chase Cancer Institute. Cells were maintained as previously described in complete RPMI 1640 medium (10% fetal calf serum, 1% penicillin-streptomycin)^23^. For 3D culture, protocols were adapted from previously published methods^15^. Briefly, Matrigel (BD Biosciences) was pipetted into the center of a 35-mm MatTek dish (P35G-0-20-C; MatTek Corp). An OVCAR5 single-cell suspension (500 μL of 1.5 × 10^4 ^cells/mL) was then placed atop the solidified Matrigel bed to settle. After 30 minutes, 2% Matrigel-supplemented complete culture media was added. 2% Matrigel-supplemented complete media was refreshed every 2 to 3 days.

### Treatment

Photodynamic therapy was chosen as this study’s primary therapeutic modality for four main reasons.: (1) PDT studies have been extensively carried out using the 3D model, such that OCT evaluation of outcome could be immediately compared to current literature^7^,^8^,^9^. (2) PDT is advantageous as the uptake and localization of therapeutic agents can be readily determined via fluorescence, allowing for one-to-one comparisons between therapeutic outcome and therapeutic uptake and delivery. (3) PDT has been found capable of reversing therapeutic resistance in ovarian cancer^27^. As therapeutic resistance is the primary reason for ovarian cancer deaths, any modality capable of reversing resistance is of great clinical and translational importance. (4) PDT is currently being employed in clinical trials for metastatic ovarian cancer, such that insight into PDT outcomes via 3D model systems would be important in interpreting clinical outcomes and planning next-step clinical trials.

(1) EtNBS and EtNBS-OH PDT

Cultures were incubated with 500 nM EtNBS or EtNBS-OH for 1.5-hour in complete medium. EtNBS-OH was synthesized in-house following previously established protocols^7^,^23^. Following incubation, the media was exchanged with fresh complete media, and each well was irradiated with 660 nm LED light (Thorlabs) at an irradiance of 100 mW/cm^2^ using a precision timed illumination setup for durations of time^15^. To evaluate combination therapy efficacy, 3D cultures were first treated with EtNBS-OH-PDT, and carboplatin treatment was then performed on the PDT-treated samples. All treatment procedures were the same as described in the above section.

(2) ALA/PpIX PDT

Cultures were incubated with 1 μM ALA (Sigma Aldrich) for 1.5 hours in complete media. Following incubation, the media was exchanged with fresh complete media and dishes were irradiated with 635 nm light at an irradiance of 50 mW/cm^2^ from a LED (Thorlabs) using the same precision timed illumination setup as above.

(3) Carboplatin

To test for dose-dependent response, cultures were treated with 200 μM carboplatin in complete media starting after ten days of growth. Cytotoxicity was measured following 72-hour incubation. Carboplatin-PDT combination combination therapy experiments were started following ten days of growth and halted on Day 14 (Days 0 through 4).

### Live/Dead Fluorescence Assay

Live/Dead fluorescence data was acquired as described previously^7^. In brief, the calcein green AM ester and ethidium homodimer were mixed and added to wells at room temperature and allowed to incubate for 40 minutes. An Olympus FV1000 confocal microscope equipped with a programmable stage was used to acquire images of both the calcein green and ethidium homodimer emission, with the pinhole opened completely. The acquired images were processed using a custom Matlab routine to calculate the normalized viability as described previously^9^.

### OCT system and data processing

The details of the OCT system have been described elsewhere^15^. Briefly, a spectral domain OCT system (Fig. 1) was developed, which includes a superluminescent diode (Superlum, HP855,) as the light source and a grating/camera (L104k, Basler) pair for detection of interferograms. The light source can provide a bandwidth of 130 nm (FWHM) with an output power of 15 mW^14^. The developed OCT system was set to acquire data at 5,000 line scans per second, providing an A scan rate of 5 kHz. The system has an axial resolution of 2.2 μm and a lateral resolution of 7.0 μm. The system SNR was determined using a glass slide to be approximately 100 dB. Images collected for this manuscript had an X dimension of 1.8 mm and a X dimension of 600 μm. 2048 A scans composed a single B scan, and 250 B scans were collected to form one volumetric image. The system camera frame rate, which is the same as the A scan rate, was 5 kHz for these experiments. At this speed, volumes were acquired in approximately 100 seconds. OCT volumes were acquired on an inverted Zeiss Axiovert 200 M microscope base equipped with a Warner Instruments incubation chamber. To maintain incubation conditions, the chamber was heated to 37 C and 5% CO_2_, balance air gas was flowed at slight positive pressure. The data acquisition software was created in-house using the LabView programming interface incorporating custom multithreaded C libraries using the Intel Performance Primitives library. The system offers stability over periods of weeks, enabling long-term, low-drift operation for monitoring treatment response.

**Figure 7:**
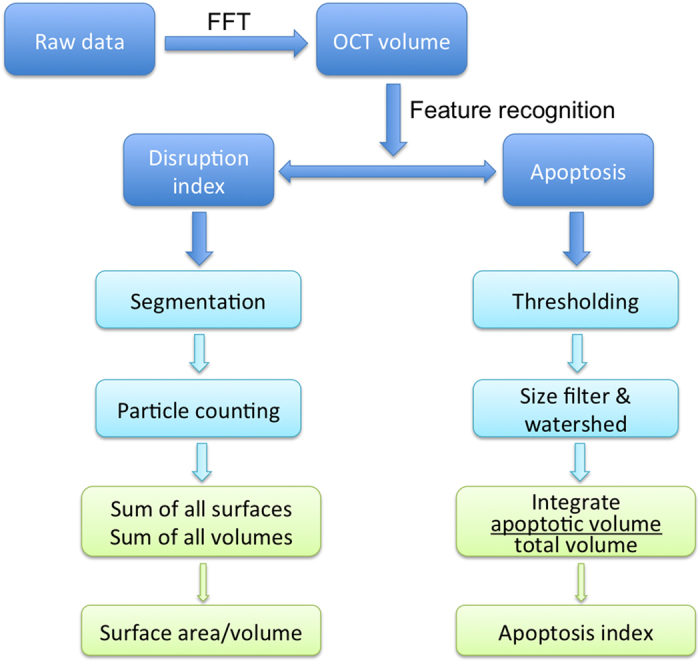
Flowchart that briefly describes how to generate surface-area-to-volume and apoptosis indices.

A flow chart that briefly describes how to generate surface-area-to-volume and apoptosis indices can be found in Fig. 7. The acquired raw data was first transformed into OCT volumes through a fast Fourier transform (FFT). Then, the analysis was divided into two methods for feature recognition. One method was developed for calculating the disruption index, which starts by segmenting and counting the individual objects within a volume followed by summing all the surfaces and volumes to generate a global surface-area-to-volume ratio. Calculated ratios are then normalized to the no-treatment control, with values then adjusted via subtraction so that zero indicates totally intact spheroids. The other method was developed to derive the apoptotic density: thresholding was determined by empirical analysis (70% higher than the average intensity of the 3D nodule) of the OCT images, then, a size filter and watershed algorithm were used to segment individual apoptotic features. Finally, the volumes of individual apoptotic features were integrated and divided by the total measured spheroid volume to calculate the apoptotic density. Detailed information regarding the thresholding and analysis method can be found in Supplementary Information.

The data analysis is first carried out for each cross-sectional xz slice. Since the volume of each slice is known based on the scanned spot size on the microscope, each segmented region yields an individual set of values. In the case of the surface-area-to-volume; each xz slice yields a set of perimeters and enclosed volumes. The total surface-area-to-volume for any data set is then simply a sum of these perimeters (for a surface) and total volume (sum of volumes). Similarly, in the case of apoptotic density, each xz slice yields a volume of apoptotic cells as well as a nodule volume. These are then summed for all slices. In this analysis, volumetric data sets yield individual parameter values; spheroids themselves are not tracked and measured individually. In this way, the analysis does not require boundary tracking and similar labels are not measured. The details on how individual cross-sectional images can be analyzed have been previously published^15^.

### Statistical Analysis

All statistical calculations were carried out using the Student’s t-test (two-tailed, unpaired, equal variance) with the JMP Pro software suite. All measurements were carried out in triplicate. Figures 2, 4 and 5 are standard box plots created with default settings using the JMP Pro software with the whiskers representing: Top: 3rd quartile + 1.5*(interquartile range); Bottom: 1st quartile − 1.5*(interquartile range). The software default also will set the whiskers to the upper and lower data point values (not outliers) if the data points in the set do not extend to the calculated ranges above.

### Image Analysis

Images for the manuscript were processed using the Fiji application. All other image analysis was carried out using custom routines written in Matlab.

## Additional Information

**How to cite this article**: Jung, Y. *et al*. Longitudinal, label-free, quantitative tracking of cell death and viability in a 3D tumor model with OCT. *Sci. Rep.* **6**, 27017; doi: 10.1038/srep27017 (2016).

## Supplementary Material

Supplementary Information
