# Supplementary material for: Longitudinal, label-free, quantitative tracking of cell death and viability in a 3D tumor model with OCT
Source: Sci Rep. 2016 Jun 1;6:27017. doi: 10.1038/srep27017 (PMC4888651; doi:10.1038/srep27017)
Supplement: Supplementary Information [file srep27017-s1.pdf]

Supplementary Information

Longitudinal, label-free, quantitative tracking of cell death and viability in a 3D tumor model with OCT

Yookyung Jung, Oliver J. Klein, Hequn Wang, Conor L. Evans

Image analysis

Determining the threshold for surface to volume ratio analysis

To find the surface boundary of the 3D cancer nodules, the raw image of OCT (8 bit grayscale image) was converted into binary image by using the Matlab command, im2bw using the graythresh function.[1] Determining proper threshold is important in this image binarization for accurate segmentation analysis. In the supplementary Fig. S1a, binary images at different thresholds are shown. Images on the top are the binary images with high threshold. The 2D boundaries of the cancer nodule at each different threshold are also overlaid on the raw OCT image in the supplementary Fig. S1b. As shown in Fig. S1a, when the threshold is scanned from the highest to lowest value, the number of segments increases. There is a distinct position where sudden increase of the segment number occurs. Red arrows indicate this position in the 'threshold-number of segments' graph and also in its derivative. (Supplementary Fig. 1c) This significant increase is due to the recognition of the background signal as segments in the binarization process. Therefore, the proper threshold was set at this distinct position. Even in situations where an intensity change of OCT image under different imaging condition was observed, 'threshold-number of segmentation' graph showed consistent pattern, and the distinct position of sudden increase could be identified.

[1] (mathworks.com/help/images/ref/graythresh.html)

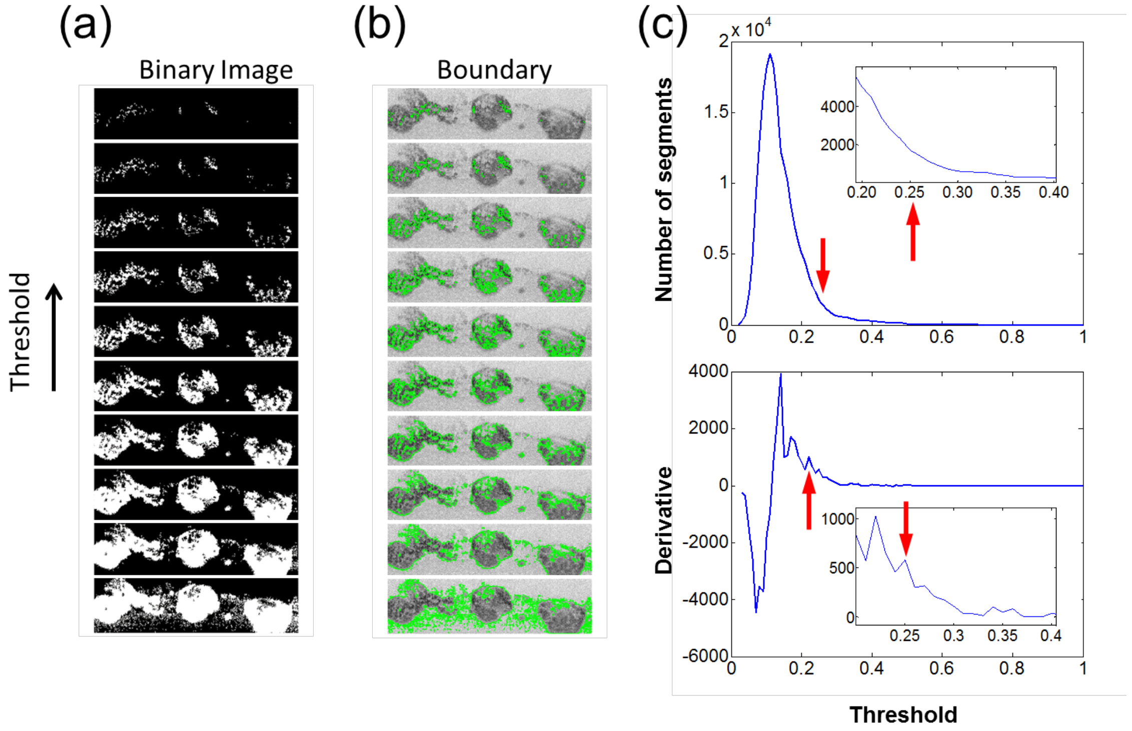

Supplementary Figure S1. Determining threshold for segmentation (a) Binary image of OCT image of 3D cancer nodule at different threshold for binarization. Bottom (top) image is acquired with low (high) threshold, respectively. (b) Boundary of the segments was overlaid on the original OCT image. (c) Graphs showing the number of segments at different threshold values and its derivative. Insets are enlarged graphs that contain the distinct position of sudden change of the number of segments. Red arrows indicate this distinct position.

Determining the threshold for apoptotic regions

The threshold to distinguish apoptotic from healthy parts of the 3D cancer nodule was determined by empirical analysis of the OCT images. The threshold for the apoptosis was set at the intensity, which is 70% higher than the average intensity of the 3D nodule. Supplementary Fig. S2a is an *en face* image reconstructed from the 3D raw OCT images. Dark contrast represents high intensity with strong light scattering, indicating apoptotic regions. Applying the intensity threshold, high intensity (apoptotic) regions are shown as red. The boundary of the 3D nodule is shown as green line (Supplementary Fig. S2b). The same 3D nodule was labeled with live/dead viability kit (ThermoFisher Scientific) and imaged using a confocal fluorescence microscope (Supplementary Fig. S2c). Green and red represent live and dead cells, respectively. The minor difference of apoptotic regions between supplementary Fig. S2b and S3c may be due to the effect of fluorescence labeling and lower resolution of the OCT compared with confocal microscopy. But convincingly, both of images show higher density of apoptotic regions at the bottom and bottom left areas. Different from the threshold for surface to volume ratio analysis, the threshold for apoptotic region was empirically determined, and may be

vulnerable to the change of OCT imaging condition. However, considering that OCT provided consistent signal level even for a week when the same imaging conditions were used, the proposed method of recognizing apoptotic regions can be very well utilized as label-free live/dead analysis tool.

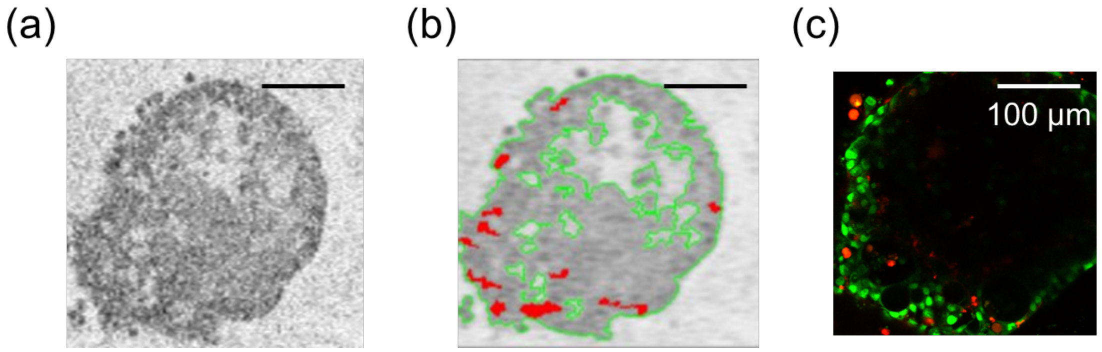

Supplementary Figure S2. One to one comparison of OCT and confocal image (a) En face OCT image of 3D cancer nodule. This image was reconstructed from the 3D raw OCT images. (b) Neighboring pixels of image (a) was averaged, and apoptotic region with high intensity (dark contrast) was indicated by red color. Boundary of the cell is drawn as green. (c) Confocal fluorescence image of the same 3D nodule. Red (green) indicates dead (alive) cells, respectively. Scale bar is 100 μm.

Representative volumetric OCT images of the 3D Spheroid Culture

Supplementary Figure S3 contains a three-dimensional plot of an OCT volume acquired from the ovarian cancer *in vitro* 3D culture. The model nodules in this image were grown for a period of thirteen days and were untreated. An inverted look-up-table is used so that the individual nodules can be visualized in the plotted 3D volume.

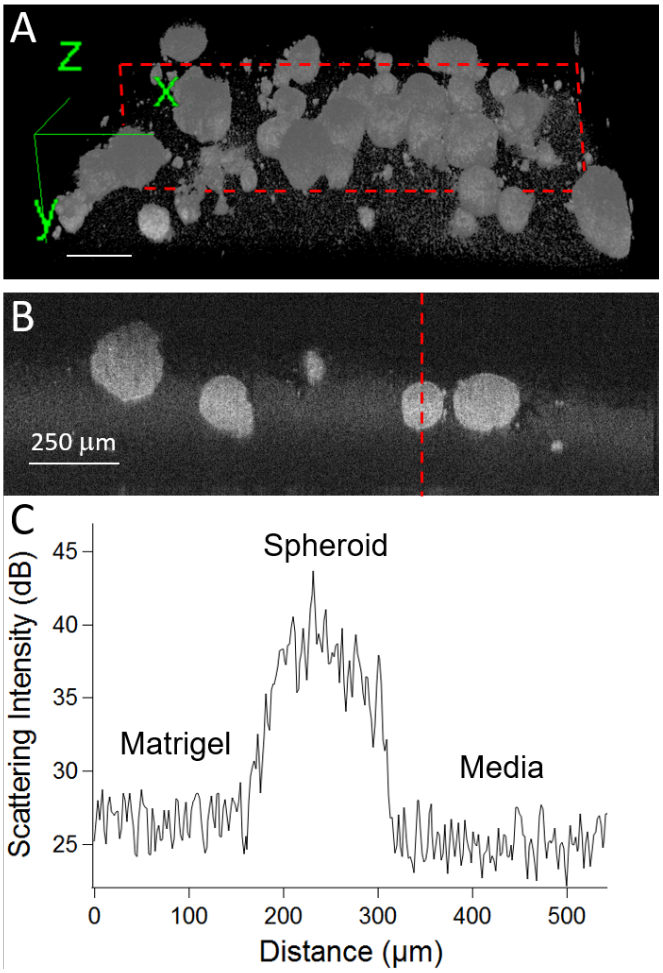

Supplementary Figure S3. Representative volumetric OCT images of a no-treatment spheroid culture's signal-to-background (a) Volumetric 3D plot of a 13-day old ovarian cancer 3D *in vitro* culture. An inverted LUT is use here so as to easily visualize individual model tumor nodules. Please note the lightly scattering Matrigel at the base of the *in vitro* culture (b) Cross-sectional image taken from the red dash line box in (a). Individual model tumor nodules can clearly be distinguished resting on top of the bed of Matrigel. (c) Plot of scattering intensity in decibels taken at the dashed red line in (b). Regions containing Matrigel, the spheroid, and the fluid growth media are noted. The scattering intensity from the spheroid is greater than 10 dB with respect to the Matrigel and media.
Scale bar = 250 μm
